# Use of Sotatercept to Facilitate Transition From Intravenous to Oral Prostacyclin Therapy

**DOI:** 10.1111/crj.70149

**Published:** 2025-12-23

**Authors:** Chebly Dagher, Maria Akiki, Kristen Swanson, Brett Carollo, Elizabeth Chernobelsky, Harrison W. Farber, Raj Parikh

**Affiliations:** ^1^ Department of Internal Medicine University of Connecticut Farmington Connecticut USA; ^2^ Division of Pulmonary, Critical Care and Sleep Hartford Hospital Hartford Connecticut USA; ^3^ Frank H. Netter MD School of Medicine at Quinnipiac University North Haven Connecticut USA; ^4^ Division of Pulmonary, Critical Care and Sleep Tufts Medical Center Boston Massachusetts USA

## Abstract

Pulmonary arterial hypertension (PAH) is a progressive disease characterized by increased pulmonary vascular resistance (PVR) leading to right ventricular failure and high mortality. Parenteral prostacyclin therapy remains the cornerstone for high‐risk patients but is limited by complications and reduced quality of life. Sotatercept, an activin signaling inhibitor, has recently emerged as an effective adjunct therapy in PAH, improving functional and hemodynamic outcomes. This case series evaluated whether the addition of sotatercept could facilitate the transition from intravenous (IV) to oral prostacyclin in eight high‐risk PAH patients who had previously failed transition attempts. All patients received background dual therapy with endothelin receptor antagonists and phosphodiesterase‐5 inhibitors and were transitioned from IV to oral treprostinil following the fifth dose of sotatercept. At 24 weeks, all eight patients successfully maintained oral therapy without re‐initiation of IV prostacyclin. Significant improvements were observed in 6‐min walk distance, WHO functional class, right ventricular systolic pressure, and PVR. No treatment discontinuations or serious adverse events occurred. These findings suggest that adjunctive sotatercept may enable safe and effective transition from parenteral to oral prostacyclin therapy, expanding treatment flexibility and improving quality of life in high‐risk PAH patients. Prospective studies are warranted to confirm long‐term outcomes.

## Graphical abstract

Sotatercept may enable successful transition from parenteral to oral prostacyclin therapy in high‐risk patients with pulmonary arterial hypertension who had previously failed transition attempts. All patients completed the transition and maintained clinical stability for 24 weeks, with notable improvements in exercise capacity, right ventricular function, and hemodynamic parameters.

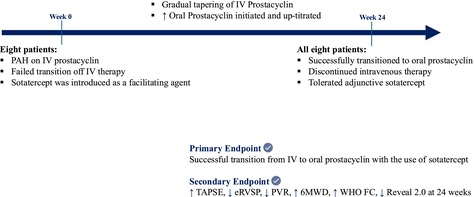

## 1 Introduction

Pulmonary arterial hypertension (PAH) is a rare disease defined by elevated pulmonary arterial pressure and increased pulmonary vascular resistance (PVR) [1]. The resulting increase in PVR imposes significant afterload on the right ventricle (RV), progressively leading to RV dysfunction, worsening symptom burden, and increased mortality risk [1].

Parenteral prostanoids are the treatment of choice for high‐risk PAH patients due to their potency [2]. They are also used as adjunctive therapy in patients with inadequate response to initial treatment [3]. Treprostinil, a prostacyclin analog, stimulates prostaglandin I2 (IP), prostaglandin D2 (DP1), and prostaglandin E2 (EP2) receptors, resulting in vasodilation and antiproliferative effects [4, 5, 6, 7]. It can be administered via subcutaneous (SC), intravenous (IV), oral, or inhaled routes [7, 8, 9]. Although highly effective, IV prostacyclin therapy is associated with risks, such as bloodstream infections and thrombosis; SC treprostinil is frequently limited by severe infusion site pain. Both IV and SC administration impose significant lifestyle restrictions due to infusion requirements [10, 11, 12, 13]. Despite the development of various transition protocols, high‐risk PAH patients often fail to transition from parenteral to oral prostacyclin, leaving them reliant on continuous infusion and its associated complications [14, 15, 16].

Recently, sotatercept, an activin signaling inhibitor (ASI), received FDA approval for the treatment of PAH following the STELLAR trial [17]. By modulating pro‐ and anti‐proliferative signaling within the pulmonary vasculature, sotatercept has demonstrated improvements in both clinical outcomes and hemodynamics when used as add‐on therapy to established PAH‐specific therapies [17]. In addition, the SOTERIA trial reported that 16.1% of patients receiving parenteral prostacyclin as background therapy were able to reduce or discontinue prostacyclin therapy after initiating sotatercept [18].

Building on these findings, we evaluated whether the addition of sotatercept could facilitate prostacyclin de‐escalation in high‐risk PAH patients who had previously failed attempts to transition from parenteral to oral therapy. In addition, we assessed multiple clinical, functional, and hemodynamic variables at baseline and at the 24‐week follow‐up.

## 2 Methods

This cohort included eight patients with PAH diagnosed via right heart catheterization (RHC), defined as a mean pulmonary artery pressure (mPAP) > 20 mmHg, pulmonary capillary wedge pressure (PCWP) < 15 mmHg, and PVR > 2 Wood units (WU). All patients were receiving IV treprostinil, Baseline demographic and clinical characteristics, including age, gender, race, PAH classification, disease duration, IV treprostinil dose, and concurrent PAH treatments, were recorded.

The primary endpoint was a successful transition from IV treprostinil to oral treprostinil after introducing sotatercept, defined as IV treprostinil discontinuation and maintenance of oral treprostinil at Week 24 while continuing sotatercept. A 24‐week follow‐up period was selected based on its established relevance in PH literature [17, 19]. The transition from IV to oral treprostinil was initiated after the fifth dose of sotatercept; this dose was chosen to ensure that sotatercept had reached its maximal effective dose and was well tolerated before initiating the transition to oral therapy [17, 20]. The target total daily dose of oral treprostinil was estimated using the equation 0.0072 × parenteral treprostinil dose (ng/kg/min) × weight (kg) [21, 22]. Hemoglobin (Hgb) and platelet counts were assessed before each sotatercept dose to monitor for potential hematologic effects. Additional variables assessed at baseline and at the 24‐week follow‐up included estimated right ventricular systolic pressure (eRVSP, mmHg), tricuspid annular plane systolic excursion (TAPSE, mm), World Health Organization (WHO) functional class, six‐minute walk distance (6MWD, meters (m)), REVEAL 2.0 risk score, and PVR (WU).

A one‐sample t‐test was performed to evaluate whether the mean changes in key clinical and hemodynamic parameters after 24 weeks were statistically significant. Given the small sample size (*n* = 8), the t‐test was chosen to compare observed sample means with hypothesized population means. Statistical significance was determined using two‐tailed tests at an alpha level of 0.05. All analyses were conducted using Python. This study was approved by the Hartford HealthCare Institutional Review Board (HHC‐2024‐0125).

## 3 Results

The cohort included eight patients with a mean age of 55.1 years (range: 33–77 years), five of whom were female. There were four patients with idiopathic PAH and four with connective tissue disease‐associated PAH (CTD‐PAH). The mean duration from PAH diagnosis was 3.1 years (range: 1–6 years). Two patients self‐identified as Asian, and six as White. All patients were receiving background PAH therapies, including endothelin receptor antagonists (ERAs) and phosphodiesterase‐5 inhibitors (PDE‐5i), and were treated with IV treprostinil therapy at baseline; mean dose of 109.4 ng/kg/min (range: 45–190 ng/kg/min) (Table 1).

**TABLE 1:** Baseline demographics and treatment characteristics of patients.

Patients	1	2	3	4	5	6	7	8
Age (years)	33	45	35	67	74	77	68	42
Gender	F	M	M	M	F	F	F	F
Race	Asian	White	Asian	White	White	White	White	White
Time since PAH diagnosis (years)	2	6	1	5	3	3	3	2
PAH classification	Group 1 Idiopathic	Group 1 Idiopathic	Group 1 Idiopathic	Group 1 Idiopathic	CTD	CTD	CTD	CTD
PAH treatment	Riociguat, Ambrisentan, IV Treprostinil	Tadalafil, Ambrisentan, IV Treprostinil	Tadalafil, Macitentan, IV Treprostinil	Tadalafil, Ambrisentan, IV Treprostinil	Tadalafil, Ambrisentan, IV Treprostinil	Tadalafil, Macitentan, IV Treprostinil	Tadalafil, Macitentan, IV Treprostinil	Tadalafil, Macitentan, IV Treprostinil

Abbreviations: CTD: connective tissue disease; F: female; IV: intravenous; M: male; ng/mn: nanograms per minute; PAH: pulmonary arterial hypertension.

Sotatercept was initiated at 0.3 mg/kg and escalated to 0.7 mg/kg by week 3 in all patients. The medication was well‐tolerated, with the most reported adverse effects being headache, nausea, and diarrhea. None of these side effects led to treatment discontinuation and no hematologic abnormalities were observed during the 24‐week period. The IV‐to‐oral prostacyclin transition followed a structured protocol, where IV treprostinil was gradually decreased while oral treprostinil was simultaneously up‐titrated in the inpatient setting [22]. The dose of IV treprostinil was reduced by up to 30 ng/kg/min per day, while the oral treprostinil dose was increased by up to 6 mg per day (2 mg TID) as tolerated [22].

All eight patients successfully transitioned from IV treprostinil to oral treprostinil, achieving the primary endpoint, and none required re‐initiation of IV therapy. Oral treprostinil dose escalation primarily occurred during the first week of exposure, though dose adjustments continued between Weeks 1 and 24. The mean total daily treprostinil dose at Week 24 was 56 mg, ranging between 26 mg and 88 mg, with most reported adverse effects being headache, nausea, diarrhea, and pain in extremities.

At 24 weeks, patients demonstrated significant improvements across clinical, functional, and hemodynamic parameters (Tables 2 and 3). The eRVSP decreased from a median of 80.5 mmHg (IQR 78.5–92.8) to 66.5 mmHg (IQR 60.5–74.3). TAPSE increased from 1.55 mm (IQR 1.4–1.7) to 1.85 mm (IQR 1.8–1.98). The WHO functional class improved from a median of 3 (IQR 3–3.25) to 1.5 (IQR 1–2). The 6MWD increased from 271 m (IQR 172–392) to 410.5 m (IQR 260–470). The REVEAL 2.0 risk score decreased from 10.5 (IQR 9–11.25) to 7 (IQR 6.75–7.5). Similarly, PVR declined from 10.15 WU (IQR 8.43–11.35) to 7.2 WU (IQR 6.55–9.35) (Tables 2 and 3).

**TABLE 2:** Comparison of clinical and hemodynamic parameters before transition and post‐transition at 24‐week follow‐up.

Patients	1	2	3	4	5	6	7	8
ERVSP (mmHg)—before transition	79	80	110	77	69	91	98	81
ERVSP (mmHg)—after transition	69	62	86	64	44	73	78	56
TAPSE (mm)—before transition	1.7	1.6	1.4	1.3	1.8	1.5	1.4	1.7
TAPSE (mm)—after transition	1.9	1.9	1.8	1.8	2.2	1.8	1.7	2.3
WHO functional class—before transition	3	3	3	4	3	3	4	3
WHO functional class—after transition	2	1	1	2	1	2	3	1
6MWD (m)—before transition	389	400	410	173	210	169	117	332
6MWD (m)—after transition	444	499	520	266	377	242	213	460
REVEAL 2.0 risk score—before transition	9	11	11	12	9	10	12	9
REVEAL 2.0 risk score—after transition	7	7	7	9	6	7	10	5
PVR (WU)—before transition	12.1	10.4	18.4	9.9	8.2	8.5	11.1	7.9
PVR (WU)—after transition	9.8	7.5	13.2	6.7	6.1	6.9	9.2	5.8

Abbreviations: 6MWT: six‐minute walk test; ERVSP: estimated right ventricular systolic pressure; m: meters; mm: millimeters; mmHg: millimeters of mercury; PVR: pulmonary vascular resistance; TAPSE: tricuspid annular plane systolic excursion; WHO: World Health Organization; WU: Wood units.

**TABLE 3:** Comparison of mean clinical and hemodynamic parameters before and after transition at 24‐week follow‐up, with mean differences and *p*‐values.

Parameter	Baseline median (IQR)	Week 24 median (IQR)	*p*
eRVSP (mmHg)	80.5 (78.5–92.8)	66.5 (60.5–74.3)	<0.001
TAPSE (mm)	1.55 (1.4–1.7)	1.85 (1.8–1.98)	<0.001
WHO Functional Class	3 (3–3.25)	1.5 (1–2)	<0.001
6MWD (m)	271 (172–392)	410.5 (260–470)	<0.001
REVEAL 2.0 Score	10.5 (9–11.25)	7 (6.75–7.5)	<0.001
PVR (WU)	10.15 (8.43–11.35)	7.2 (6.55–9.35)	<0.001

Abbreviations: 6MWT: six‐minute walk test; ERVSP: estimated right ventricular systolic pressure; m: meters; mm: millimeters; mmHg: millimeters of mercury; PVR: pulmonary vascular resistance; TAPSE: tricuspid annular plane systolic excursion; WHO: World Health Organization; WU: Wood units.

## 4 Discussion

In this cohort, we initiated sotatercept in eight patients with high‐risk PAH features, as defined by the 2015 ERS/ESC guidelines (table 13) [23], who were receiving parenteral treprostinil and had previously failed attempts to transition to oral prostacyclin therapy. Sotatercept was used as a therapeutic adjunct to facilitate this transition from IV to oral therapy. Notably, all eight patients successfully transitioned to oral prostacyclin and tolerated the therapy for 24 weeks without requiring reinitiation of parenteral therapy.

Previous studies have consistently shown that baseline hemodynamics strongly predict the success of transitioning PAH patients from parenteral to oral prostacyclin therapy, with lower success rates observed in those with more severe hemodynamic compromise [24, 25, 26, 27, 28, 29]. Moreover, a multicenter study by Chakinala et al. demonstrated that transition is feasible in carefully selected, low‐risk PAH patients [16]. In contrast, our cohort consisted of high‐risk patients with compromised baseline hemodynamics, making them poor candidates for transition under standard criteria (Table 2). Their prior failed transition attempts highlight the challenge in this population, where transition is traditionally unsuccessful. However, sotatercept, which received FDA approval following the STELLAR trial, has been shown to improve various clinical, functional, and disease‐related outcomes in PAH patients [17]. In addition, in the SOTERIA trial, among 163 patients receiving parenteral treprostinil at baseline, nine were successfully transitioned to oral treprostinil and one to inhaled treprostinil after initiation of sotatercept. Notably, 16.1% of participants were able to discontinue or de‐intensify prostacyclin therapy, further supporting its role as a therapeutic adjunct in facilitating the transition from parenteral to oral prostacyclin therapy in our high‐risk PAH patients [18]. Also, the Phase 3 ZENITH trial evaluated the addition of sotatercept to maximum tolerated background therapy in high‐risk PAH patients, defined by WHO functional class 3 or 4, REVEAL 2.0 risk score ≥ 9, similar to the high risk features observed in our cohort [30]. Data released thus far suggest the trial demonstrated a significant reduction in morbidity and mortality compared to placebo, further supporting the use of sotatercept in our patient cohort [30].

Additionally, a recent single‐center analysis evaluating withdrawal of prostacyclin‐pathway agents after initiation of sotatercept provides important context for our findings [31]. In that study, discontinuation of selexipag was generally well tolerated, but attempts to withdraw parenteral treprostinil were successful in only about one‐third of patients and were often accompanied by a decline in cardiac output and rise in PVR, with several patients requiring dose escalation or re‐initiation of therapy [31]. In contrast, our approach did not attempt full withdrawal but instead used sotatercept to support a structured transition from IV to oral treprostinil. These contrasting strategies highlight why our outcomes diverged from prior reports.

Notably, our case series differs from prior studies on successful transition, as it included high‐risk patients in whom the addition of sotatercept likely facilitated transition and also led to significant improvements at 24‐week follow‐up. This included significant improvements in mean 6MWD, WHO functional class, eRSVP and PVR at 24 weeks, aligning with the benefits observed in the PULSAR [20] and STELLAR [17] trials after initiation of sotatercept. Additionally, the REVEAL 2.0 risk score decreased, reinforcing the clinical stability achieved post‐transition. While previous transition to oral therapy trials primarily included low‐risk patients, our findings suggest that sotatercept may enhance transition feasibility even in some high‐risk PAH patients.

Beyond the clinical benefits, transitioning to oral therapy also offers practical advantages by eliminating the inconvenience and complications associated with parenteral prostacyclin therapy [7, 8, 9, 10, 11, 12, 13]. Continuous IV or SC administration requires permanent central venous access or infusion pumps, which increase the risk of bloodstream infections, thrombosis, and infusion site pain [10, 11, 12, 13]. The successful transition of all eight patients in our cohort allowed them to discontinue parenteral therapy, potentially improving quality of life, reducing treatment burden, and minimizing infection risk. Given these benefits, our findings highlight the potential role of sotatercept in expanding transition eligibility, not only to improve clinical outcomes but also to make treatment more convenient for high‐risk PAH patients.

While PAH treatment strategies have traditionally focused on therapy escalation, there is limited guidance on when and how to de‐escalate treatment in PH patients. Our case series suggests that sotatercept may facilitate the transition from parenteral to oral therapy in high‐risk PAH patients, aligning with findings from the SOTERIA trial, which demonstrated that some patients were able to discontinue or de‐intensify parenteral prostacyclin after initiating sotatercept. These findings highlight the need for formalized guidelines on de‐escalation strategies in PAH. Establishing clear criteria for identifying patients suitable for therapy reduction could help optimize long‐term disease management.

This case series is limited by its small sample size, single‐center design, and short follow‐up period, which may not fully capture the long‐term outcomes of transition. While our findings are descriptive rather than definitive, they provide insight into the potential role of sotatercept in facilitating transition and highlight the need for further studies in larger, diverse populations.

## 5 Conclusion

This case series provides provocative evidence that sotatercept may facilitate the transition from parenteral to oral prostacyclin therapy in high‐risk PAH patients who had previously failed transition attempts. Notably, all patients successfully transitioned and maintained clinical stability for 24 weeks, with significant improvements in exercise capacity, right ventricular function, and hemodynamics following transition. These findings suggest that adjunctive sotatercept may expand transition eligibility beyond traditionally stable patients, potentially reducing the long‐term need for parenteral therapy. While these results are promising, larger, prospective studies with extended follow‐up are needed to further evaluate the durability of transition success and long‐term clinical outcomes in this population.

## Author Contributions

Conceptualisation, C.D. and R.P.; methodology, C.D. and R.P.; validation, H.W.F. and R.P.; formal analysis, C.D.; investigation, C.D., M.A., and R.P.; resources, R.P.; data collection, K.S., B.C., and E.C., data curation, C.D.; writing − original draft preparation, C.D.; writing − review and editing, H.W.F., R.P., M.A., K.S., and B.C.; visualisation, C.D.; supervision, H.W.F., and R.P.; project administration, R.P.

## Funding

The authors have nothing to report.

## Conflicts of Interest

The authors declare no conflicts of interest.

## Data Availability

Data sharing not applicable to this article as no datasets were generated or analysed during the current study.
